# Supplementary figures and images for: Light-harvesting chlorophyll a/b-binding protein-coding genes in jatropha and the comparison with castor, cassava and arabidopsis
Source: PeerJ. 2020 Jan 28;8:e8465. doi: 10.7717/peerj.8465 (PMC6993755; doi:10.7717/peerj.8465)

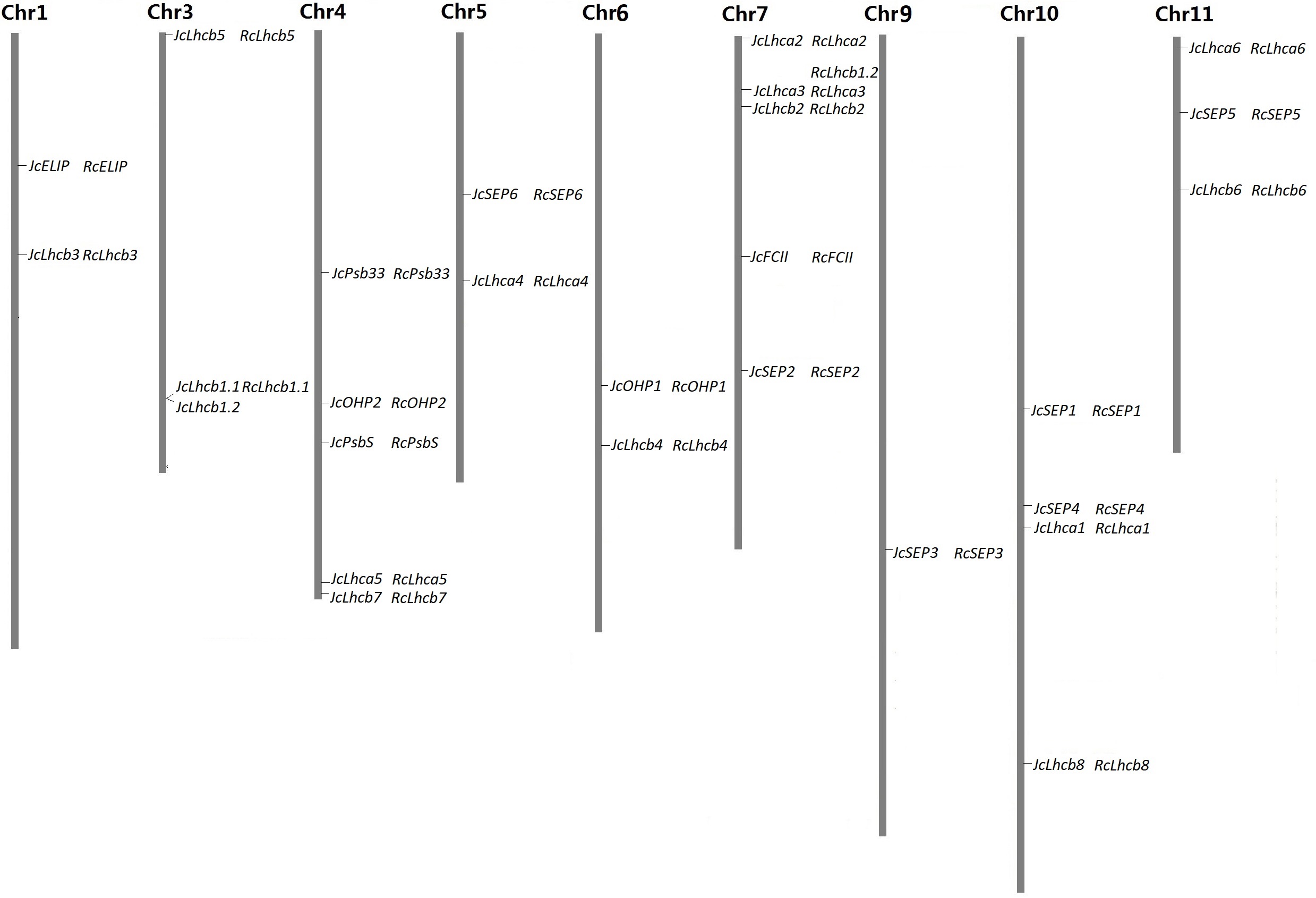

Supplement: Figure S1 — The chromosome serial numbers are indicated at the top of each chromosome, and 27 RcLhc superfamily genes are shown just behind their collinear genes in jatropha (where RcLhcb1.2 with no collinear gene in jatropha). (Chr: chromosome; Jc: Jatropha curcas; Rc: Ricinus communis). [file peerj-08-8465-s001.jpg]

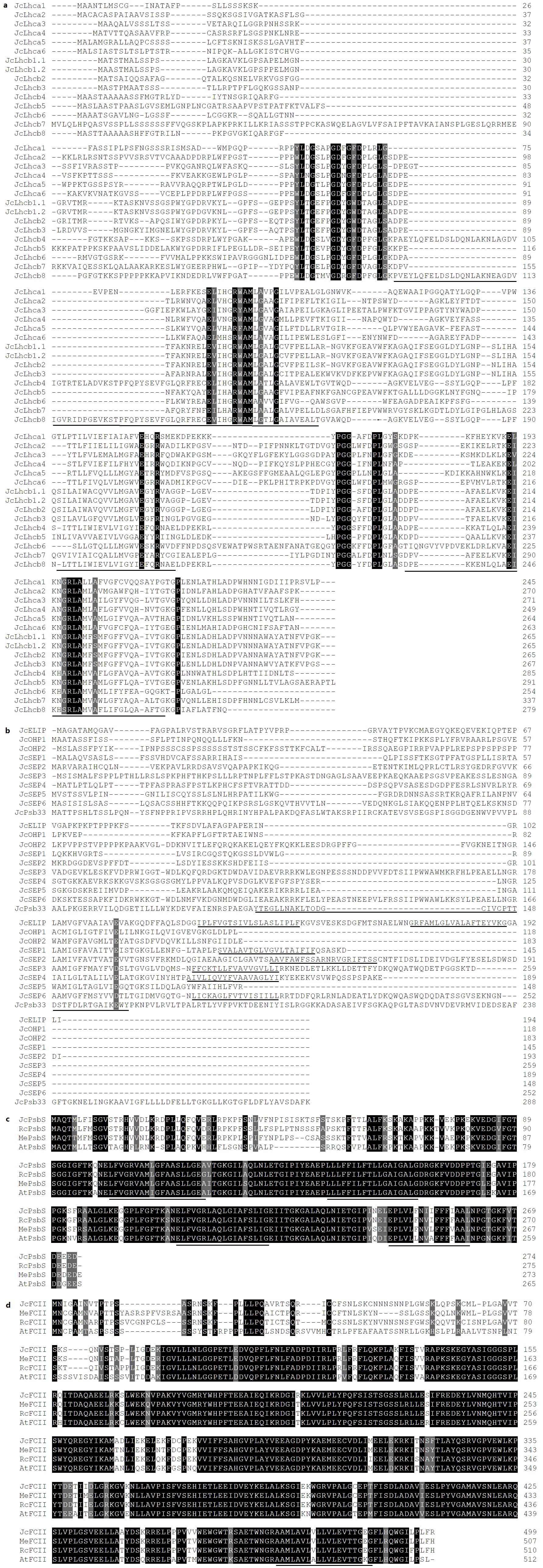

Supplement: Figure S2 — (A) Sequence alignment of the JcLhc family; (B) Sequence alignment of the JcLil family; (C) Sequence alignment of JcPsbS with RcPsbS, MePsbS, and AtPsbS; (D) Sequence alignment of JcFCII with RcFCII, MeFCII, and AtFCII. Sequence alignment was performed using MUSCLE and predicted TMHs were underlined. (TMH: transmembrane helix). [file peerj-08-8465-s002.jpg]

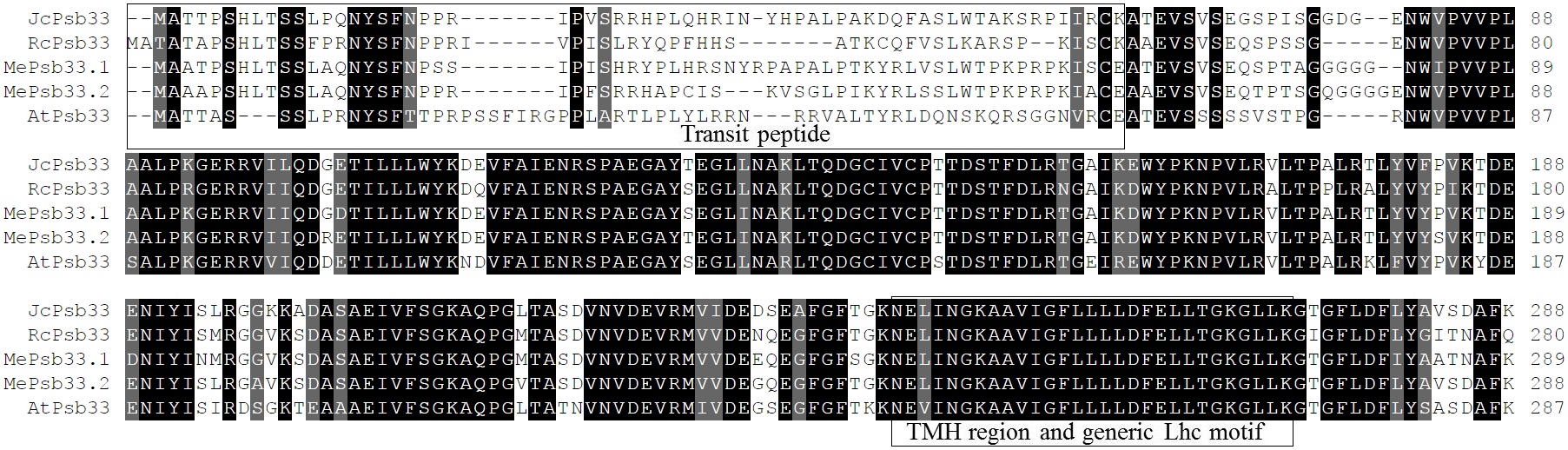

Supplement: Figure S3 — Sequence alignment was performed using MUSCLE. The predicted chloroplast transit peptide and Lhc motif-bearing TMH region were boxed. (TMH: transmembrane helix). [file peerj-08-8465-s003.jpg]
